# Suppression of Homologous Recombination by insulin-like growth factor-1 inhibition sensitizes cancer cells to PARP inhibitors

**DOI:** 10.1186/s12885-015-1803-y

**Published:** 2015-10-29

**Authors:** Oreekha Amin, Marie-Claude Beauchamp, Paul Abou Nader, Ido Laskov, Sanaa Iqbal, Charles-André Philip, Amber Yasmeen, Walter H. Gotlieb

**Affiliations:** 1 Division of Gynecologic Oncology, Jewish General Hospital, McGill University, 3755 Cote Ste. Catherine Road, Montreal, H3T 1E2 QC Canada; 2 Segal Cancer Center, Lady Davis Institute of Medical Research, McGill University, 3755 Cote Ste. Catherine Road, Montreal, H3T 1E2 QC Canada; 3 Department of Oncology, McGill University, Montreal, QC Canada

**Keywords:** Ovarian cancer, BRCA1, Homologous recombination, Insulin-like growth factor 1, PARP

## Abstract

**Background:**

Impairment of homologous recombination (HR) is found in close to 50 % of ovarian and breast cancer. Tumors with *BRCA1* mutations show increased expression of the Insulin-like growth factor type 1 receptor (IGF-1R). We previously have shown that inhibition of IGF-1R results in growth inhibition and apoptosis of ovarian tumor cells. In the current study, we aimed to investigate the correlation between HR and sensitivity to IGF-1R inhibition. Further, we hypothesized that IGF-1R inhibition might sensitize HR proficient cancers to Poly ADP ribose polymerase (PARP) inhibitors.

**Methods:**

Using ovarian and breast cancer cellular models with known *BRCA1* status, we evaluated their HR functionality by RAD51 foci formation assay. The 50 % lethal concentration (LC50) of Insulin-like growth factor type 1 receptor kinase inhibitor (IGF-1Rki) in these cells was assessed, and western immunoblotting was performed to determine the expression of proteins involved in the IGF-1R pathway. Moreover, IGF-1R inhibitors were added on HR proficient cell lines to assess mRNA and protein expression of RAD51 by qPCR and western blot. Also, we explored the interaction between RAD51 and Insulin receptor substance 1 (IRS-1) by immunoprecipitation. Next, combination effect of IGF-1R and PARP inhibitors was evaluated by clonogenic assay.

**Results:**

Cells with mutated/methylated *BRCA1* showed an impaired HR function, and had an overactivation of the IGF-1R pathway. These cells were more sensitive to IGF-1R inhibition compared to HR proficient cells. In addition, the IGF-IR inhibitor reduced RAD51 expression at mRNA and protein levels in HR proficient cells, and sensitized these cells to PARP inhibitor.

**Conclusion:**

Targeting IGF-1R might lead to improved personalized therapeutic approaches in cancer patients with HR deficiency. Targeting both PARP and IGF-1R might increase the clinical efficacy in HR deficient patients and increase the population of patients who may benefit from PARP inhibitors.

## Background

Ovarian cancer is the most lethal gynecologic malignancy. Despite the fact that 70–80 % of ovarian cancer patients initially respond to standard treatments, most will relapse and ultimately die of the disease [1]. Having reached a stage of stagnation with conventional chemotherapeutic agents, there is a desperate need for new therapeutic modalities to overcome the persistent/recurrent tumor cells.

Further, primary triple negative breast cancer (TNBC), which are defined by the lack of expression of estrogen receptors, progesterone receptors and HER2 gene amplification and overexpression, represent approximately 16 % of all breast cancers and exhibit poor clinical outcome due to aggressive behavior and lack of effective therapies [2].

Up to 30 % of ovarian cancer and TNBC patients show functional impairment of *BRCA1/2* genes [3, 4] and women carrying *BRCA1/2* germline mutations are at an increased risk of developing ovarian and breast cancer [5–8]. These mutations in *BRCA1/2* genes exhibit impaired cellular ability to repair double-stranded DNA breaks via the homologous recombination (HR) repair pathway, leading to reduced RAD51 foci formation following DNA damage [9, 10]. Moreover, in cancer cells with loss of function of proteins involved in HR including BRCA1/2, but also RAD51, ATM or ATR, Poly (ADP-ribose) polymerase (PARP) inhibition, which interferes with single stranded DNA repair, has been shown to induce specific cancer cell killing, called synthetic lethality [11].

*BRCA1* has been shown to directly affect the IGF-1R pathway [12] and studies have suggested that *BRCA1/2* deficient breast cancer cells are associated with elevated expression of Insulin like growth factor-1 receptor (IGF-1R) [13–15]. IGF-1R are widely expressed on normal and neoplastic cells [13, 16–20], and an IGF-1 autocrine loop was described in ovarian and breast cancer cells [21–23]. Inhibition of the IGF-1 pathway suppresses ovarian cancer cell survival *in vitro* [22, 24, 25] and *in vivo* in xenograft models [26], and its expression is associated with cancer progression [17, 27]. Moreover, IGF-1 promotes proliferation and survival of TNBC cells [28], and is involved in tumor metastasis and invasion [29–31], increasing the appeal of targeting the IGF-1R pathway. Finally, an association between inhibition of the IGF-1R and suppression of the HR DNA repair pathway has been described in prostate cancer [32] and non-small cell lung cancer cells exposed to radiation [33]. In this study, we evaluate the interactions between HR and IGF-1R inhibition and whether IGF-1R inhibition can sensitize cells to PARP inhibitors through HR suppression.

## Methods

### Cells lines

The epithelial ovarian cancer cell lines SKOV3, UWB1.289 (ATCC, Manassas, VA, USA), IGROV1 (NCI), OVCAR8 (Biomiga, San Diego, CA USA) were used in this study. SKOV3, IGROV1, OVCAR8 were grown in RPMI-1640 medium supplemented with 10 % fetal bovine serum (FBS), 2 mM glutamine, and 10 μg/ml gentamicin and UWB1.289 was grown in 50 % MEGM medium (supplemented with hEGF, BPE, insulin, hydrocortisone), 50 % RPMI-1640 (supplemented with 10 % FBS, 2 mM glutamine) and 10 μg/ml gentamicin. The breast cancer cell lines BT20, MDA-MB-231, MDA-MB-436, HCC1937 were obtained from ATCC, Manassas, VA, USA. SUM149PT cell line was obtained from Asterand, Detroit, MI, USA. BT20 and MDA-MB-231 were grown in DMEM supplemented with 10 % FBS, and 10 μg/ml gentamicin. MDA-MB-431 and HCC1937 were grown in RPMI-1640 medium supplemented with 10 % FBS, and 10 μg/ml gentamicin. SUM149PT was grown in RPMI-1640 medium supplemented with 10 % FBS, 10 μg/ml gentamicin and growth factors (insulin, hydrocortisone). According to published data, the *BRCA1* gene profile status of these cells is as follow: SKOV3, BT20, MDA-MB-231 (wild type *BRCA1* gene); IGROV1 (heterozygous 280delA *BRCA1* mutation); OVCAR8 (carrying methylated *BRCA1* gene); UWB1.289 (homozygous 2594delC *BRCA1* gene mutation), MDA-MB-436 (homozygous 5396 + 1G > A *BRCA1* mutation); HCC1937 (homozygous 5382insC *BRCA1* mutation); SUM149PT (homozygous 2288delT *BRCA1* mutation) [34, 35]. Each cell line was passaged every 4 to 6 days. All cells were maintained at 37 °C in a 5 % CO_2_, 95 % air atmosphere incubator. All assays were performed in the respective cell medium.

Patient tumor-derived ovarian cancer cells labeled GOC31, GOC17, GOC15, GOC13, GNOV1, GOC23 were isolated in our laboratory from six surgical specimens, all from high grade (grade 3) stage 3/4 serous ovarian cancer. This study was approved by the ethic committee of Jewish General Hospital (JGH) and all patients participating in this study gave informed consent in accordance with the JGH ethics committee regulations (protocol#03-041). Two of the epithelial cell lines (GOC23 and GNOV1) were derived from patients carrying the 5385insC *BRCA1* germline mutations. Presence of the mutations in these cell lines was confirmed by the molecular pathology department. Primary cell lines were grown in OSE medium supplemented with 20 % FBS and growth factors (insulin, EGFR, hydrocortisone, BPE). The cells were routinely passaged every 4 to 6 days. All cells were maintained at 37 °C in a 5 % CO_2_, 95 % air atmosphere incubator. All assays were performed in the respective cell medium.

### Survival assays

The clonogenic assay was used to determine survival fraction of cells [36]. Briefly, 350–800 cells were plated in 6-well flat bottom cell culture plates in duplicates. Twenty-four hours after plating, cells were washed and fresh medium was added in the presence or absence of increasing doses of IGF-1Rki (BMS-536924) and PARP inhibitor (olaparib) alone and in combination. Media containing the drug was refreshed on day 4. Colonies were fixed and stained after 7 days of treatment with 1.5 ml of 6.0 % glutaraldehyde and 0.5 % crystal violet and colonies were counted using the GelCount, Optronix. The surviving fraction (SF) of cells was calculated as follows: $\mathrm{S}\mathrm{F}=\frac{Number\  of\  colonies\  formed\  after\  treatment\ }{Number\  of\  cells\  seeded\ x\  Plating\  Efficiency\ }$, where $\mathrm{Plating}\ \mathrm{Efficiency}=\frac{Number\  of\  colonies\  formed\  in\  control}{Number\  of\  cells\  seeded}$ [36]. The interaction between IGF-1Rki and PARP inhibitor was assessed using the multiple drug effects analysis method of Chou and Talalay [37]. This method quantitatively describes the interaction between two or more drugs, with values less than 1 indicating synergistic interactions, values greater than 1 indicating antagonistic interactions, and values equal to 1 indicating additive interactions.

The Alamar Blue assay was used to determine cell viability. Monolayers of 2000 cells were plated into 96-well flat-bottom cell culture plates in triplicates. Twenty-four hours after plating, when the cells had attached and reached ~40 % confluency, cells were washed and the medium was replaced with medium containing 1 % FBS with increasing doses of IGF-1Rki for 72 h. Controls included equal amount of DMSO. Cell viability was assessed by visual inspection of the plates and by using the AlamarBlue colorimetric assay. AlamarBlue (Invitrogen, Burlington,Ontario) assay allows quantitative analysis of cell viability via the innate metabolic activity that results in a chemical reduction of AlamarBlue that changes from the oxidized (blue) form to the reduced (pink) form. After cells were treated, 6 μl of AlamarBlue was added into each well. When the color of the dye changed (approximately 4 h), plates were read in an ELISA plate reader at 2 different wavelengths, 562 nm and 620 nm to plot the graph. Percentage of reduced AlamarBlue was calculated using the following equation: Reduced AlamarBlue % = (A562 − A620) × Rо; where A562 and A620 are sample absorbencies minus the media blank; $\mathrm{R}o=\frac{AO562}{AO620}$, where AO562 is the absorbance of oxidized form at 562 nm, and AO620 is the absorbance of oxidized form at 620 nm.

#### Immunofluorescence analysis

Cells were seeded in 6-well plates at a density of 1 × 10^5^ cells / well on a sterile coverslip. Twenty-four hours after plating, when the cells had attached and reached ~60 % confluency, cells were washed and the medium was replaced with medium containing 1 μg/ml cisplatin for 1 h and allowed to recover for ~6 h. In another setting, the cells were treated with medium containing 5 μM IGF-1Rki for 24 h, followed by 1 μg/ml cisplatin treatment for 1 h and allowed to recover for ~6 h. The cells were then washed in phosphate-buffered saline (PBS) and fixed using 4 % formaldehyde. They were subsequently permeabilized with 0.2 % Triton-X 100 in PBS for 15 min. After blocking with 2 % BSA/PBS for 1 h at room temperature, cells were incubated with primary antibodies: RAD51 (Santa Cruz Biotechnology, CA, USA; 1:200) in blocking buffer for 60 min at room temperature. Cells were then washed in PBS and incubated with AlexaFlour 488chicken anti-rabbit IgG secondary antibody (Invitrogen, CA, USA; 1:500) for 30 min. Finally, cells were counterstained with 4′, 6-diamidino-2-phenylindole (DAPI) for 5 min before the final wash and photographed using LEICA; DMI6000B microscope.

#### Protein extraction and western blot analysis

Cells were lysed in radioimmunoprecipitation assay (RIPA) buffer (25 mM Tris∙HCl, pH 7.6, 150 mM NaCl, 1 % NP-40, 0.25 % sodium deoxycholate, 0.1 % SDS) supplemented with protease inhibitor cocktail tablet and phosphatase inhibitor tablet (PhosphoSTOP, Roche Diagnostics, Mannheim, Germany). Total protein content was measured according to Pierce BCA protein assay kit (Thermo Scientific, Rockford, IL, USA). Then protein lysates (50 μg) were resolved electrophoretically on denaturing SDS-polyacrylamide gels, and transferred to 0.45 μm nitrocellulose membranes. After blocking in 5 % milk in PBST, membranes were probed with the following primary antibodies: anti-mouse BRCA1(Ab-1) (Calbiochem), anti-mouse p-ATM (Ser1981) (Millipore), anti-rabbit ATM (D2E2), anti-rabbit p-IGF-1R beta (Y1135/1136), anti-rabbit IGF-1R beta (111A9), anti-rabbit p-IRS1(S612), anti-rabbit IRS1, anti-rabbit p-AKT(S473), anti-rabbit AKT, anti-rabbit p-S6 (Ser235/236), anti-rabbit S6 (5G10), anti-rabbit beta-actin (Cell signalling, Danvers, MA ,USA) and anti-rabbit RAD51 (Santa Cruz Biotechnology, Dallas,Texas, USA). Immunobloted proteins were visualized using horseradish peroxidise (HRP)-conjugated secondary antibodies and antigen-antibody complexes were detected using the ECL system.

#### Immunoprecipitation analysis

Clarified protein lysates (300–500 μg/ml) were pre cleared with 25 μl of protein G-magnetic beads (EMD Millipore, ON, Canada), followed by 1 h incubation at 4 °C. Magnetic field was applied for 30 s to pull beads to the side of the tube and supernatant was pipetted to a clean tube. Then 1–5 μg of antibody; anti-rabbit RAD51 (Santa Cruz Biotechnology) or anti-rabbit IRS-1 (D23G12) (Cell signalling), was added to crude cell lysate, followed by overnight incubation at 4 °C. The next day, 25 μl of protein G magnetic beads suspension was added and incubated for 2 h at 4 °C. Then magnetic field was applied and supernatant was removed and discarded. Beads pellet was washed with 500 μl of RIPA buffer by gentle vortex. Again magnetic field was applied and supernatant was removed and discarded (RIPA buffer wash was repeated 2 more times). Beads pellet was then resuspended in 25 μl of 2X SDS Sample Loading Buffer and incubated at 95 °C for 10 min. After centrifuge, magnetic field was applied to sample, and supernatant was loaded on SDS-PAGE gel for electrophoresis. Separated proteins were transferred to membranes. Membranes were then probed with a specific antibody followed by peroxidase-conjugated appropriate secondary antibody and visualization by ECL.

### shBRCA1 transfection

SKOV3 cells were seeded in 6-well flat-bottom cell culture plates at a density of 0.25x10^6^cells / well. Lipofectamine (Invitrogen, Burlington, Ontario, Canada) (1:1) was mixed with control shRNA and BRCA1 shRNA separately in RPMI-1640 with no FBS. Following 30 min of incubation at room temperature, both negative and BRCA1 shRNA were added to their respective wells. The cells were incubated at 37 °C for 5 h. Pools of stably transfected cells were selected using 2 mg/ml puromycin for up to a week.

### Gene expression analysis

SKOV3 and BT20 cells were treated with IGF-1Rki (1–5 μM) for 12 h, 16 h and 24 h time points. RNA was isolated from cells using Quick-RNA Mini prep kit (Zymo research, CA, USA). cDNA was synthesized using M-MLV retrotranscriptase enzyme. Template cDNA was added to Maxima SYBR Green/ROX qPCR master mix (2X) (Thermo Scientific, MA,USA) with RAD51 and 36B4 primers. Quantification of gene expression was performed using the Applied Biosystems 7500 fast real-time PCR system (life technologies).

#### Statistical analysis

Statistical analysis was performed using Prism and the non-parametric two-tailed paired T-Test. P < 0.05 was considered statistically significant.

## Results

### Determination of BRCA1 expression in cancer cells

**Fig. 1:**
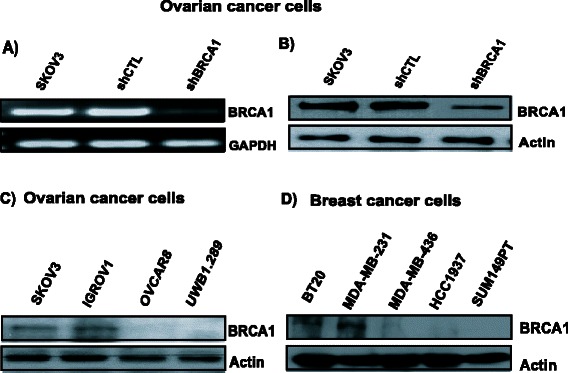
BRCA1 expression in ovarian and breast cancer cells. Reduced expression of *BRCA1* was observed in SKOV3 transfected with shBRCA1: **a**) mRNA and **b**) protein levels using RT-qPCR and western blot analysis, respectively. BRCA1 protein expression status in **c**) ovarian and **d**) breast cancer cells, using western blot analysis. Results shown are one representative experiment out of three independent experiments

SKOV3 cells transfected with shBRCA1 were assessed by RT-PCR and western blot. As shown in Fig. 1a and b, we found reduced levels of *BRCA1* mRNA and protein in the transfected SKOV3 cell line. We next evaluated BRCA1 protein expression in ovarian and breast cancer cells, as shown in Fig. 1c,d and confirmed previously published data where OVCAR8, UWB1.289, MDA-MB436, HCC1937 and SUM149T are BRCA1-deficient [34, 35].

### Decreased HR functionality in cancer cells having low/absent BRCA1 protein expression

**Fig. 2:**
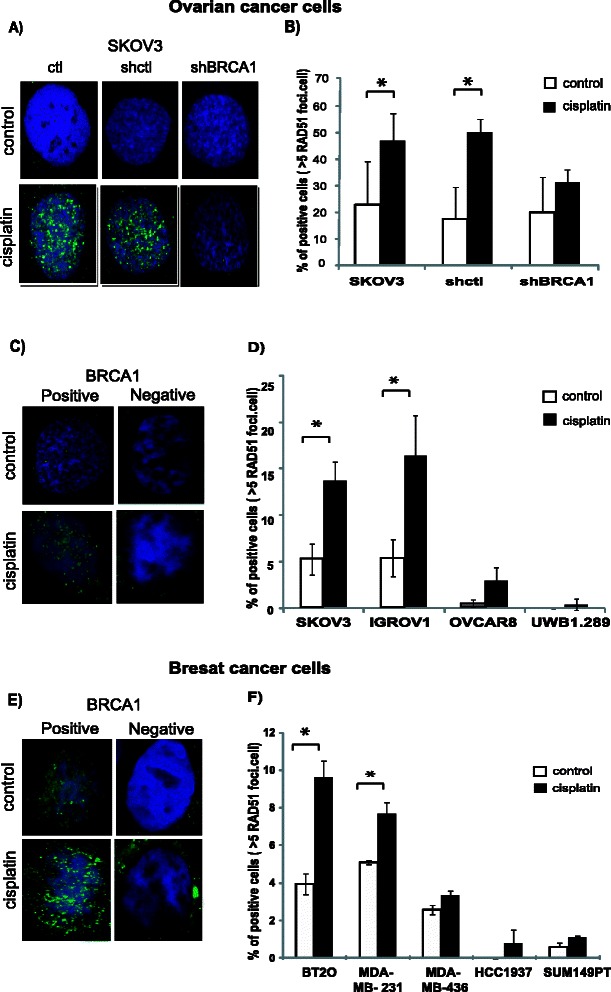
Reduced RAD51 foci formation in cancer cells with low/absent BRCA1 expression. Cells were treated with 1ug/ml cisplatin for one hour, allowed to recover for six hours and then fixed for immunofluorescence. Immunofluorescence staining images of RAD51 foci in ovarian (**a**, **c**) and breast (**e**) cancer cells with respect to the BRCA1 protein expression are shown at 100X magnification. Quantitative representation of the percentage of cells with positive RAD51 foci is shown in fig (**b**, **d**, **f**). Cells with >5 foci/nucleus were considered positive. Results represent the average of three independent experiments. *p < 0.05

We then assessed the correlation of BRCA1 protein expression and HR functionality in these cells. To assess HR functionality, we evaluated the RAD51 foci formation upon DNA damage with one hour treatment of 1 μg/ml cisplatin, by immunofluorescence, as an indication of cells’ ability to repair DNA double strand break [38]. We observed the reduction in level of nuclear RAD51 foci formation after cisplatin treatment in cells having low or absent BRCA1 protein expression, as shown in Fig. 2a-f, suggesting a lower capacity of DNA repair through HR.

**Fig. 3:**
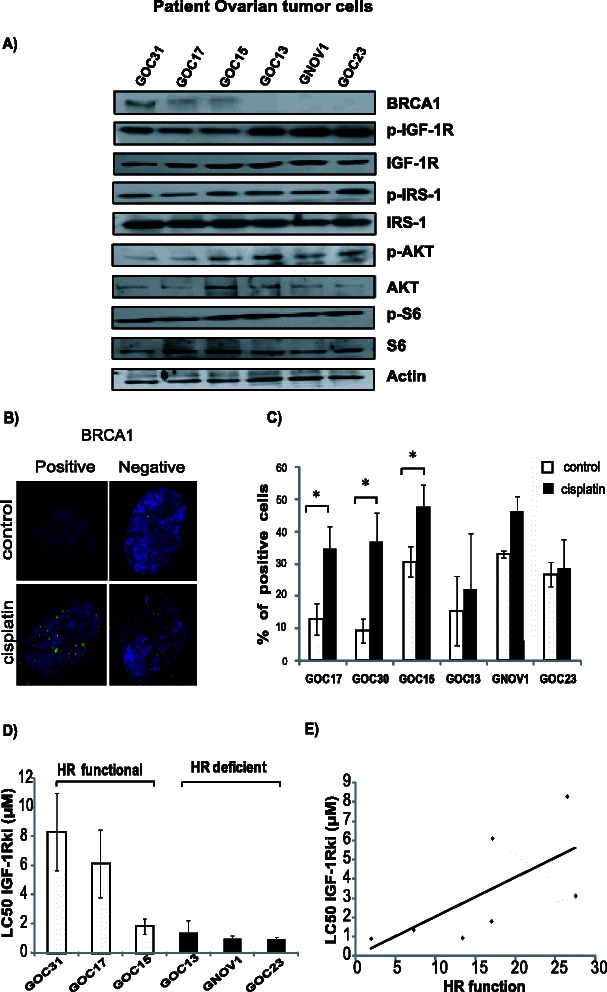
HR deficient ovarian cancer cell lines derived from patients show higher sensitivity to IGF-1Rki. Over activation of IGF-1R pathway was observed by western blotting in cells having no BRCA1 expression (**a**). HR functionality was determined using RAD51 foci (green) formation following cisplatin treatment. A representative image (magnification 100X) is shown in (**b**), and quantification in (**c**) (cells with >5 RAD51 foci/nucleus were considered positive). **d** LC50 of the IGF-1Rki was determined in these cells using the Alamar survival assay **e**) Correlation was assessed between the LC50 of IGF-1Rki and the HR functionality of cells. The HR functionality was determined by the difference between the % of cells forming RAD51 foci after cisplatin treatment and the control. All the results represent the average of three independent experiments. *p value <0.05

We performed similar experiments in patient-derived ovarian cancer cells. Among the six cell lines tested, three had no detectable BRCA1 levels (Fig. 3a). The cells that did not express BRCA1 had reduced RAD51 foci formation upon DNA damage, suggesting a deficient HR functionality (Fig. 3b,c).

### Overactivation of IGF1-R pathway in cancer cells having deficient HR

**Fig. 4:**
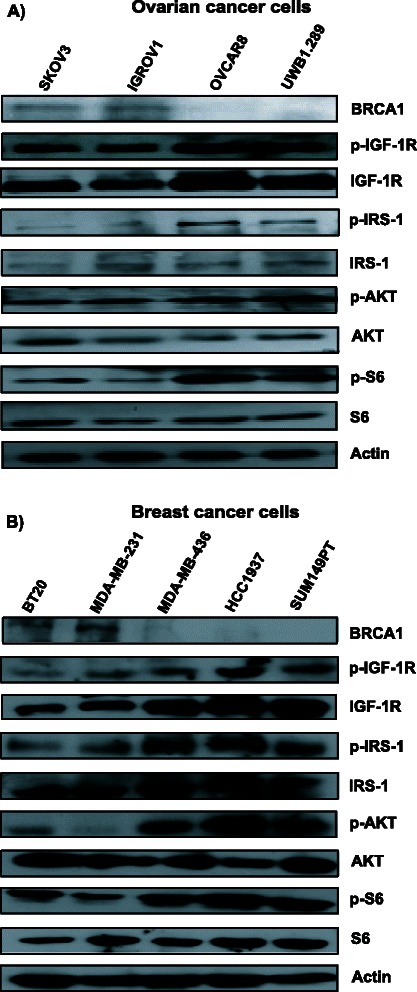
IGF-1R pathway is over expressed in HR deficient cancer cells. Cellular lysates from **a**) ovarian and **b**) breast cancer cells were subjected for western blot analysis for the indicated proteins involved in the IGF-1R pathway. One representative blot out of three is shown

It was reported that *BRCA1* deficient breast tumors have higher IGF-1R expression [13, 39]. We thus assessed the expression of downstream proteins of the IGF-1R pathway in the breast and ovarian cancer cells. As shown in Fig. 4a,b, higher levels of p-IGF-1R, IGF-1R, p-IRS-1, p-AKT, and p-S6 was observed in cells lacking BRCA1 protein expression. Similarly, we have found higher levels of p-IGF-1R, p-IRS-1, p-AKT and p-S6 in patient derived ovarian tumor cells without BRCA1 expression, as shown Fig. 3a.

### Increased sensitivity of cancer cells bearing HR deficiency to an IGF-1Rki

**Fig. 5:**
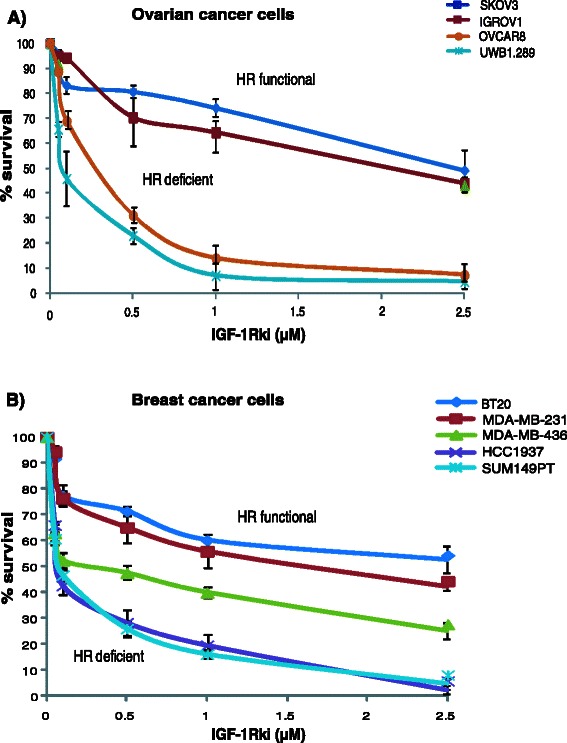
Increased sensitivity of HR deficient cells to IGF-1Rki. Clonogenic survival assay was performed in the presence of increasing doses of IGF-1Rki in ovarian (**a**) and breast (**b**) cancer cells. Results represent the average of four independent experiments

We next evaluated the sensitivity of these cells to IGF-1Rki by survival assays. Ovarian and breast cancer cells with functional HR displayed a 40–60 % decrese in cell survival at a dose of 2.5 μM. In comparison, cells with low or absent BRCA1 expression, and with a concurrent HR deficiency, were found to be more sensitive, showing a survival of only 10–20 % at a dose of 1 μM, as demonstrated in Fig. 5a,b.

Similar results were observed with patient-derived ovarian cancer cells, i.e. increased sensitivity to IGF-1Rki of cells having low HR functionality as compared to cells capable of forming increased level of RAD51 foci formation (Fig. 3d). We next evaluated if a correlation existed between the LC50 IGF-1Rki and the HR cellular function. Based on the RAD51 foci formation data, we plotted the LC50 of IGF-1Rki against the difference between the percentage of cells forming RAD51 foci (>5 foci/ cell) with cisplatin treatment and control (no cisplatin treatment). This ‘x’ value was used as an indication of the extent of HR functionality for each cell line. As shown in Fig. 3e, a tendency towards a positive correlation was observed between LC50 IGF-1Rki and HR functionality of cells, again suggesting that HR-deficient cells are more sensitive to IGF-1Rki.

### IGF-1R inhibition impacts Homologous Recombination

**Fig. 6:**
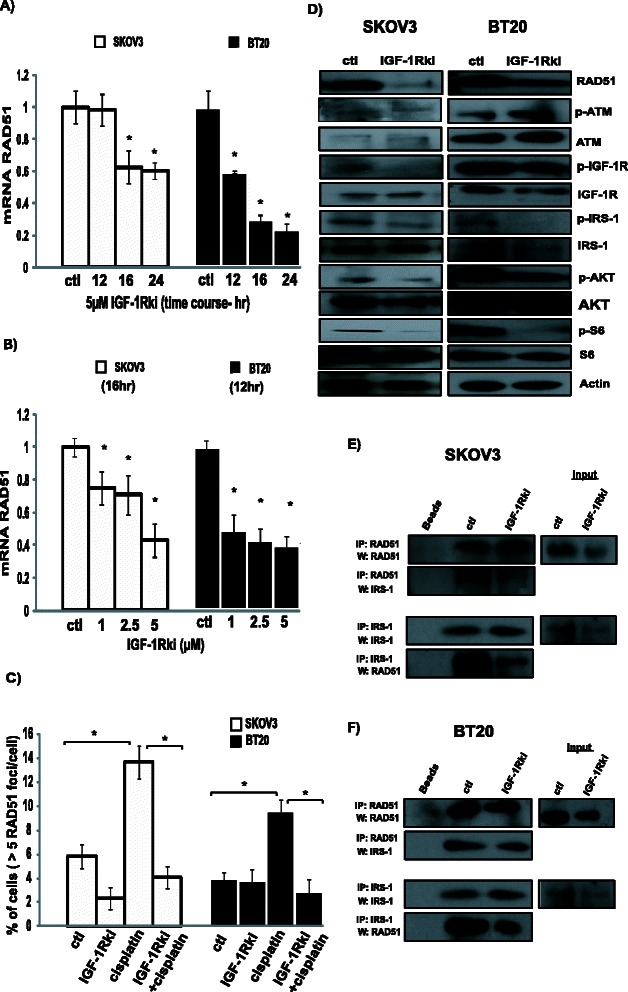
IGF-1R inhibition reduced RAD51 expression both at mRNA and protein levels. Using HR proficient SKOV3 and BT20 cancer cells, quantitative RT-PCR analysis showed the reduction of RAD51 mRNA levels in cells treated with IGF-1Rki (**a**) at different times (12 h-24 h) (**b**) and doses (1-5 μM). **c** IGF-1R inhibition decreases cisplatin-induced RAD51 foci formation in these cells (>5 RAD51 foci/nucleus were considered positive). **d** Treatment with 5uM of IGF-1Rki for 24 h reduces the expression of RAD51 and IGF-1R pathway proteins, determined by Western blot analysis. A representative blot out of 3 is shown. **e** After treatment with 5uM IGF-1Rki for 24 h, clarified protein lysates from SKOV3 (**e**) and BT-20 (**f**) cells were subjected to immunoprecipitation. Precipitates were blotted against RAD51 and IRS-1. One representative blot out of 4 is shown. Results represent the average of four independent experiments. *p value <0.05

We next evaluated the effect of IGF-1Rki on HR by assessing the expression RAD51, a crucial player in HR DNA repair. Interestingly, we observed a reduction of RAD51 mRNA levels at 16 h in SKOV3 and at 12 h in BT20 cells. We also observed a reduction of RAD51 mRNA levels in a dose–response manner (Fig. 6b). Next, using RAD51 foci formation assay, we found that the cells treated with the 5 μM IGF-1Rki showed decreased formation of RAD51 foci after induction of DNA damage with 1 μg/ml cisplatin treatment, as compared to cells treated with 1 μg/ml cisplatin treatment alone, as shown in Fig. 6c. This reduction was probably due to a decrease in RAD51 protein levels in response to IGF-1Rki, as shown in Fig. 6d, suggesting that IGF-1R inhibition directly impacts HR functionality in cancer cells. Furthermore, phosphorylated proteins of the IGF-1R pathway were determined as positive controls in SKOV3 and BT20 cells. We found that p-IGF-1R, p-IRS1, p-AKT and p-S6 protein levels were decreased in the IGF-1Rki treated cells as compared to the untreated cells as shown in Fig. 6d. Next, we determined if the protein interaction between IRS-1 and RAD51 in our cancer cells was modified after IGF-1Rki treatment using immunoprecipitation. As shown in Fig. 6e, levels of IRS-1 associated with RAD51 and vice versa were significantly reduced in cells treated with IGF-1Rki. Taken together, these data suggest that IGF-1Rki suppresses RAD51 expression and thus affects directly HR DNA repair in cells.

### HR deficiency induced by IGF-1Rki sensitizes cancer cells to PARP inhibition

**Fig. 7:**
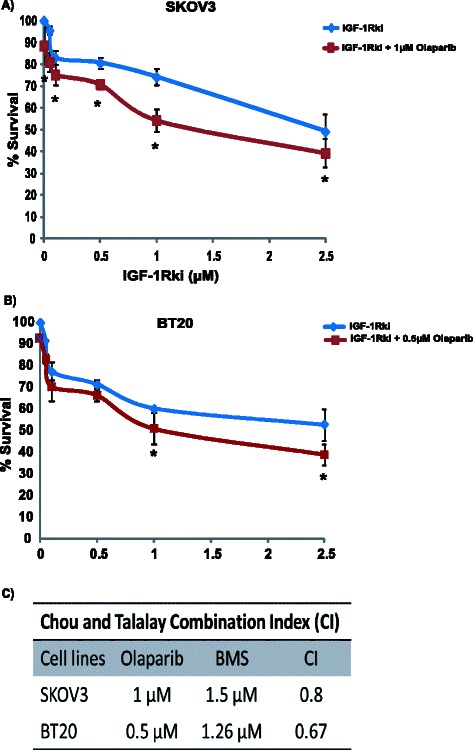
IGF-1Rki and PARP inhibitor combination effect in HR Proficient cancer cells. SKOV3 (**a**) and BT-20 (**b**) cancer cells were treated with increasing doses of IGF-1Rki in the presence of sublethal doses of 1uM (**a**) or 0.5uM (**b**) olaparib for 7 days and survival was determined using the clonogenic assay. **c** The combination index was calculated where CI < 1 indicates synergy between the two drugs. Data represents the average of four independent experiments. *p value <0.05

It was reported that HR deficient cells are sensitive to inhibition of PARP [40]. We evaluated whether HR impairment provoked by IGF-1R inhibition confered increased sensitivity to PARP inhibition. As shown in Fig. 7a,b, we treated SKOV3 and BT20 cells with increasing doses of IGF-1Rki (0.01–5 μM) and PARP inhibitor (Olaparib 0.5–5 μM), alone and in combination. We found decreased survival of cells with combination treatment as compared to IGF-1Rki and PARP inhibitor alone. Further, to determine the nature of the interaction between IGF-1Rki and PARP inhibitor we used the multiple drug effects analysis method of Chou and Talalay (see Materials and Methods) [37]. Interestingly, in both cell lines tested, we observed the combination treatment to be synergistic, as mentioned in Fig. 7c. The Combination Index (CI) for SKOV3 was 0.8 (Olaparib 1 μM and BMS 1.5 μM) and 0.67 for BT20 (Olaparib 0.5 μM and BMS 1.26 μM).

## Discussion

*BRCA1/2* germline mutation carriers are at an increased risk of developing ovarian and breast cancer [6, 7, 41–43]. *BRCA1* is a transcription factor involved in numerous cellular processes, including DNA damage repair, and has been shown to directly interact with IGF signaling such that variants in this pathway may modify risk of cancer in women carrying *BRCA* mutations [12]. Transcriptional suppression of the IGF-1R gene by *BRCA1* has also been reported in breast and endometrial cancer, and loss-of-function mutation of *BRCA1* leads to amplification [44–46] and constitutive activation of the IGF-1R pathway in breast cancer [13–15]. The data presented in this manuscript suggests an interaction between the two pathways, as demonstrated by the enhanced protein levels of IGF-1R, p-IGF-1R, p-IRS-1, p-AKT and p-S6 in ovarian and breast cancer cells having loss-of-function mutations of *BRCA1*.

We found an increased sensitivity to IGF-1R inhibition in cells having loss-of function mutations of *BRCA1* (HR deficient) as compared to wild type *BRCA1* (HR proficient) cells, with a positive correlation between LC50 of the IGF-1Rki and HR functionality. This data is consistent with the finding that suppression of IRS-1 inhibits the growth of *BRCA1*-deficient tumor cells [47], and that HR deficient prostate cancer cells (mutated/methylated BRCA1) are more sensitive to IGF-1R inhibition [48].

Despite the strong rationale around IGF-1R pathway inhibition, the promising preclinical data and its reasonable tolerability, the clinical efficacy has been disappointing [49, 50]. Our study shows that cells with HR deficiency are more sensitive to IGF-1Rki, suggesting that this subset of patients could benefit from this targeted therapy. In order to identify these patients, a clinically relevant test is needed. HR functionality was previously assessed in ascites of ovarian cancer patients and breast tumor biopsy samples by RAD51 ionizing radiation induced foci assay, and represent a potentially useful way of functional diagnostic testing to identify patients with HR deficiency [38, 51]. These patients could then be selected to be treated with IGF-1Rki therapy, with a higher likelihood of responding.

Moreover, we found that IGF-1Rki affects HR in ovarian and breast cancer cells by reducing the expression of RAD51 at the mRNA level, and this subsequently decreased RAD51 protein level and its interaction with IRS-1. This is consistent with the described increased HR by IGF-1R activation associated to the translocation of RAD51 to the sites of damaged DNA (nuclear foci) [52]. To date, the role of IGF-1R inhibition in DNA repair has been reported in several studies [32, 48, 53, 54], emphasizing the role of IGF-1R inhibition in suppression of HR, but the mechanism is not fully known. In prostate cancer cells, co-inhibition of epidermal growth factor receptor and IGF-1R reduced phosphorylation of IRS-1 and its interaction with RAD51, suppressing HR and increasing radio-sensitivity [53].

Our data further suggests that IGF-1R inhibition suppresses HR by downregulation of RAD51, and that this allows a strong antitumor activity in combination with PARP inhibitors in *BRCA1* wild-type (HR proficient) ovarian and breast cancer cells. This is consistent with our previous work demonstrating that inhibition of the IGF-1R sensitizes ovarian cancer cells to PARP inhibition [24]. Similar sensitization to PARP inhibition in TNBCs without BRCA mutations were described with PI3K blockade [55]. These findings could help improve the clinical activity of the PARP inhibitors in non BRCA mutant tumors.

Several factors limit the interpretation of our results. First, we used commercial cell lines that differ from patient tumors which are often heterogeneous. In addition, *in vitro* results should be interpreted cautiously as the effective concentration delivered to the cell can vary from *in vivo* models, and the effects are evaluated without the interactions with the *in vivo* microenvironment. Further steps include xenograft models that will provide the rationale to investigate the clinical efficacy of dual PARP and IGF-1R inhibition in cancer cells, an approach that could expand the subset of patients who may benefit from PARP inhibitors.

## Conclusions

Treatment options are limited for patients with high grade ovarian cancer and metastatic triple negative breast cancer. Recent work has suggested a role for BRCA1/2 and defective HR in sporadic ovarian cancer resulting from somatic mutations or epigenetic mechanisms. Here, we assessed the correlation between IGF-1R inhibition and HR functionality of breast and ovarian cancers cells, and observed an increased sensitivity of HR deficient cancer cells to IGF-1Rki. Moreover, our data suggest that inhibiting the IGF-1R pathway suppresses HR by reduction in expression of RAD51. We further showed that IGF-1Rki and PARP inhibitors act in synergy to inhibit cancer cells. Here, we show that IGF-1R blockade results in HR impairment and sensitization to PARP inhibition in ovarian and breast cancer cells without *BRCA* mutations, providing a rationale to combine IGF-1R and PARP inhibitors in this indication and providing new opportunities for the development of targeted personalized cancer therapy, expanding the number of patients that could benefit from PARP inhibitors.
